# The Attenuated Protective Effect of Outer Membrane Vesicles Produced by a *mcr-1* Positive Strain on Colistin Sensitive *Escherichia coli*

**DOI:** 10.3389/fcimb.2021.701625

**Published:** 2021-07-28

**Authors:** Xue Li, Lang Sun, Congran Li, Xinyi Yang, Xiukun Wang, Xinxin Hu, Tongying Nie, Youwen Zhang, Xuefu You

**Affiliations:** Beijing Key Laboratory of Antimicrobial Agents, Institute of Medicinal Biotechnology, Chinese Academy of Medical Sciences & Peking Union Medical College, Beijing, China

**Keywords:** outer membrane vesicles, *mcr-1*, colistin resistance, lipid A, protection

## Abstract

Resistance to colistin, especially mobilized colistin resistance (mcr), is a serious threat to public health since it may catalyze a return of the “pre-antibiotic era”. Outer membrane vesicles (OMVs) play a role in antibiotic resistance in various ways. Currently, how OMVs participate in *mcr-1*-mediated colistin resistance has not been established. In this study, we showed that both OMVs from the *mcr-1* negative and positive *Escherichia coli* (*E. coli*) strains conferred dose-dependent protection from colistin. However, OMVs from the *mcr-1* positive strain conferred attenuated protection when compared to the OMVs of a *mcr-1* negative strain at the same concentration. The attenuated protective effect of OMVs was related to the reduced ability to absorb colistin from the environment, thus promoting the killing of colistin sensitive *E. coli* strains. Lipid A modified with phosphoethanolamine was presented in the OMVs of the *mcr-1* positive *E. coli* strain and resulted in decreased affinity to colistin and less protection. Meanwhile, *E. coli* strain carrying the *mcr-1* gene packed more unmodified lipid A in OMVs and kept more phosphoethanolamine modified lipid A in the bacterial cells. Our study provides a first glimpse of the role of OMVs in *mcr-1* -mediated colistin resistance.

## Introduction

Infections caused by multidrug-resistant (MDR) Gram-negative bacteria have led to the rebirth of the “last-line” antibiotic colistin. However, a gradual increase in colistin resistance has been reported in the past few years (Cai et al., 2012; Ah et al., 2014; Monaco et al., 2014; Bialvaei and Samadi Kafil, 2015; El-Sayed Ahmed et al., 2020), which is becoming a great challenge for clinical practice. Thus, understanding the mechanism of resistance to colistin and developing new therapeutic strategies against colistin-resistant superbugs are of great significance.

Colistin belongs to the amphipathic ‘detergent-like’ polypeptide antibiotics. The bactericidal action of colistin involves initial electrostatic binding to lipopolysaccharide (LPS) in the outer membrane of Gram-negative bacteria, with the resultant displacement of Ca^2+^ and Mg^2+^, followed by the insertion of the fatty acyl chain within the outer membrane leading to the leakage of intracellular contents (Falagas et al., 2005; Biswas et al., 2012; Cai et al., 2015). The most identified mechanism of resistance to colistin in Gram-negative bacteria involves modifications to the LPS which consists of the O antigen, the core oligosaccharide and the lipid A moiety (Nikaido, 2003). In *E. coli*, modification of lipid A with 4-amino-4-deoxy-L-arabinose, phosphoethanolamine (pEtN) and/or galactosamine results in a reduced negative charge of LPS and reduced interaction between colistin and the LPS (Breazeale et al., 2005; Chen and Groisman, 2013; Moffatt et al., 2019). While the genes involved in most of these modifications are chromosomally encoded, the first mobilized colistin resistance gene (*mcr-1*) conferring plasmid-mediated resistance was reported in 2016 and poses a great threat in colistin resistance dissemination globally (Liu et al., 2016; Ling et al., 2020). So far, 10 *mcr* genes (*mcr-1*–*mcr-10*) have been identified in *Enterobacteriaceae* (Wang et al., 2020). The MCR-1 enzyme is attached to the inner-membrane of Gram-negative bacteria (Gao et al., 2016) and belongs to the phosphoethanolamine (pEtN) transferase enzyme family. In *E. coli*, expression of the *mcr-1* gene results in the addition of phosphoethanolamine to lipid A and resistance to colistin (Ling et al., 2020).

Outer membrane vesicles (OMVs) are spherical structures (20–300 nm) derived from the cell envelope of Gram-negative bacteria. OMVs have been found to mediate a variety of functions, including delivery of virulence factors (Bonnington and Kuehn, 2014), secretion of misfolded proteins to relieve cell envelope stress (McBroom and Kuehn, 2007), and resistance to antimicrobials (Chattopadhyay and Jagannadham, 2015). OMVs can help bacteria to fight against antimicrobials either by horizontally transferring antibiotic-resistant genes or directly absorbing the antibiotic from the environment (Schwechheimer and Kuehn, 2015). Previous research has shown that the addition of OMVs provided immediate resistance to colistin in *E. coli* and *Pseudomonas syringae* (Manning and Kuehn, 2011; Kulkarni et al., 2014; Kulkarni et al., 2015). However, the underlying molecular mechanisms have not been fully elucidated. More importantly, little is known about the role of OMVs in MCR-mediated colistin resistance. Therefore, this study set out to assess the role of OMVs in colistin resistance mediated by *mcr-1* and to investigate the underlying molecular mechanisms.

In this work, OMVs isolated from *E. coli* strains carrying the *mcr-1* gene, as well as control strains without the *mcr-1* gene, were investigated for their ability to protect against colistin. The ability of OMVs to bind colistin was evaluated. The lipid A contents were characterized and analyzed to investigate the mechanisms of protection against colistin. Our data, for the first time, showed how OMVs were involved in *mcr-1* related colistin resistance.

## Materials and Methods

### Strains and Culture Conditions

*E. coli* 08-85 was isolated from the blood of a patient and carried the *mcr-1* gene on the plasmid (Liang et al., 2021). This clinical pathogenic strain and *E. coli* ATCC25922 selected as an *mcr-1* negative control strain, were used in the study. In our previous work, *E. coli* K12 was constructed with *mcr-1* which is transcriptionally fused to a constitutive promoter on the chromosome. This isogenic strain (*E. coli* 50434), as well as *E. coli* K12, was also used in the study. The transcriptional expression level of *mcr-1* of *E. coli* 50434 was 102.6 ± 66.05 (compared with housekeeping genes *cysG* and *hcaT*) and resistant to colistin with the minimal inhibitory concentration (MIC) of 4 μg/ml (Qiao, 2019), while the MIC of colistin against *E. coli* K12 was 1 μg/ml. *E. coli* ATCC25922 and *E. coli* K12 were purchased from the American Type Culture Collection (ATCC, VA, USA), and all bacterial strains used in this study were stored in the Collection Center of Pathogen Microorganism of Chinese Academy of Medical Sciences (CAMS-CCPM-A, China). Strains used in this study are listed in **Table 1**.

**Table 1:** Strains used in this study.

Strains	Source	*mcr-1*	MIC of Colistin	MBC of Colistin	Ref
*E. coli* ATCC25922	ATCC	−	1	2	–
*E. coli* 08-85	Human, blood	+	4	8	(Liang et al., 2021)
*E. coli* K12 (ATCC47076)	ATCC	−	1	2	(Qiao, 2019)
*E. coli* 50434	Recombineered from K12	+	4	8	(Qiao, 2019)

All strains were cultivated in LB broth (Difco, NJ, USA) or grown on an LB agar plate. The log-phase culture was obtained by starting with a 1:1,000 dilution of overnight culture and growing to OD_600nm_ of 0.8 with shaking at 220 rpm, 37°C.

### Purification of OMVs

OMVs were isolated from the log-phase culture of *E. coli* 25922, *E. coli* 08-85, *E. coli* K12, and *E. coli* 50434 according to the procedure published previously (Chutkan et al., 2013; Klimentová and Stulík, 2015). Briefly, 500 ml of LB broth was inoculated with 0.5 ml of overnight culture and incubated at 37°C with shaking at 220 rpm with or without colistin (2 μg/ml) until OD_600nm_ reached 0.8. Cells were pelleted by centrifugation (4,700 rpm for 30 min), and the supernatant was filtered through a 0.22-μm membrane (Millipore, MA, USA). The filtrates were concentrated to 10 ml using an Amicon^®^ stirred cell (Millipore, MA, USA) with the membrane of 100 kDa cutoff. The concentrated filtrates were ultracentrifuged (125,000 × g) for 1 h at 4°C. The pellets were resuspended in phosphate buffered saline (PBS).

The OMV preparations were checked for the presence of bacterial cells by plating the vesicle suspension on LB agar. The total protein concentration in the purified OMV preparations was examined by Bradford Coomassie assay (Thermo Fisher, MA, USA), and the OMV concentrations used in subsequent assays refer to this protein-based value.

### Transmission Electron Microscopy

The OMVs were resuspended in PBS and negatively stained with 2% phosphotungstic acid solution (pH 7.3). The OMVs were then fixed with 2.5% glutaraldehyde overnight at 4°C. Samples were washed, dehydrated, and placed on copper grids for examination by transmission electron microscope (JEOL, Japan).

### Nanoparticle Tracking Analysis

The OMV samples were serially diluted to make the final concentration within the measurement range in particle-free PBS (filtered through a 0.1-μm membrane). The measurement of OMV diameter was performed using ZetaView (Particle Metrix, Germany) following the instructions of the manufacturer.

### OMV-Mediated Protection Assay

For the protection of growth, an overnight culture of *E. coli* ATCC25922 or *E. coli* K12 was diluted 1:1,000 with LB broth and treated with colistin (2 μg/ml) alone or simultaneously with OMVs isolated from *E. coli* ATCC25922, *E. coli* 08-85, *E. coli* K12, or *E. coli* 50434 at the indicated concentrations (20, 2, or 0.2 μg/ml). A 350-μl aliquot was added to each well of a honeycomb plate (Oy Growth Curves Ab Ltd, Finland) in triplicate. The turbidity was read every 15 min using a Bioscreen C analyzer (Oy Growth Curves Ab Ltd, Finland). The mean optical density at each time point was calculated. The experiment was repeated independently twice.

For protection in the killing assay, 1 ml of log-phase culture (OD_600nm_ = 0.8) of *E. coli* ATCC25922 or *E. coli* K12 was treated with 2 μg/ml colistin alone, or simultaneously with colistin and OMVs at the same concentration used in the Bioscreen assay. *E. coli* ATCC25922 or *E. coli* K12 alone was used as a positive control. Cultures were incubated at 37°C for 6 h and plated on LB agar to determine the colony-forming units (CFUs).

### BODIPY TR Cadaverine Displacement Assay

To determine if OMVs were able to bind to BC and quench its fluorescence, the fluorescence titration of OMVs was performed based on a previous assay (Wood et al., 2004). BC was diluted in 50 mM Tris-HCl (pH 7.4) to make a final concentration of 4 μM. The BC-OMV solutions were prepared by the addition of aliquots of stock solutions of OMVs into BC solutions, of which the final concentration of OMVs ranged from 0.1 to 4 μg/ml. A 100 μl of BC-OMV solution was added into a non-binding surface (NBS) black 96 well microplate (Corning, NY, USA) in triplicate. The fluorescence intensity was read with excitation at 580 nm and emission at 620 nm (Enspire 2300, PerkinElmer, MA, USA).

The displacement of OMV-bound BC by colistin was performed as previously described (French et al., 2019) with slight modifications. Briefly, 100 μl of 50 mM Tris-HCl (pH 7.4) buffer containing 4 μM BC, 2 μg/ml OMVs and serially diluted colistin (0.02 to 200 μg/ml) was added into NBS black 96 well microplate (Corning), and the fluorescence intensity was read.

### Lipid A Isolation and LC-MS/MS Characterization

Isolation of lipid A was performed according to the protocol of Henderson et al. (2013). The OMV pellets and bacterial cells were resuspended in 1.6 ml of PBS, and 2 ml of chloroform and 4 ml of methanol were added for a single-phase Bligh-Dyer (chloroform: methanol: water; 1:2:0.8 v/v). The samples were lysed for 20 min at room temperature, and pellets were collected (2,000 × g for 20 min) and washed with single phase Bligh-Dyer. The pellets were resuspended in 3.6 ml of mild acid hydrolysis buffer (50 mM sodium acetate, pH 4.5; 1% SDS), assisted by sonication at a constant duty cycle for 5 s at 25% output, and incubated at 95°C for 1 h. Lipid A was then extracted by adding 4 ml of chloroform and 4 ml of methanol for a two-phase Bligh-Dyer (chloroform:methanol:water, 2:2:1.8 v/v). The lower phase was collected, washed twice with neutral upper phase, and dried in a rotational vacuum centrifuge.

An U3000 system (ThermoFisher, MA, USA) coupled to a Triple TOF 5600 mass spectrometry (AB sciex, WA, USA) was employed in the study. The extracted lipid A was eluted using a gradient that consists of buffer A (MeOH/ACN/H_2_O, 1:1:1, 5 mM NH_4_OAC) and buffer B (MeOH/IPA, 1:4, 5 mM NH_4_OAC), which started from 10% buffer B for 1 min, 1–6 min to 60% buffer B, 6–18 min to 100% buffer B, then kept at 100% buffer B for 2 min, followed by equilibration with 10% B for 5 min at a flow rate of 0.5 ml/min. ZORBAX RRHD 300Å SB C8 (4.6 × 150 mm, 5 µm, Agilent, CA, USA) was maintained at 40°C. The extracted lipids were dissolved in dichloromethane-methanol (2:1, vol/vol) and infused into the ion source at 5 to 10 μl/min. Lipid A was analyzed in negative mode at 4,500 V with a mass range from 50 to 2,400 m/z. The relative quantitation of lipid A species was determined by calculating the peak area with MultiQuant 3.0. The percent composition was calculated as the peak area of each lipid A species divided by the sum of all lipid A species.

### Quantification of OMV Production

To quantitate OMV yield, log-phase culture (OD_600nm_ = 0.8) of *E. coli* K12 and *E. coli* 50434 growing with or without colistin (2 μg/ml) was harvested for OMV isolation, and a portion of the culture was aliquoted and plated on LB agar to determine CFUs. The lipid content in OMV preparations was measured with the lipophilic dye FM4-64 (Molecular Probes, OR, USA) as described previously (McBroom et al., 2006). In brief, OMVs were incubated with FM4-64 (3.3 μg/ml) for 10 min at 37°C. The negative controls were set as OMVs alone and FM4-64 alone. The fluorescence intensity was measured using a PerkinElmer Enspire 2300 with excitation and emission at 506 nm and 750 nm respectively.

### Statistical Analysis

Statistical analysis was performed with GraphPad Prism 8 software. *P*-values were calculated by t-test, one-way ANOVA or two-way ANOVA followed by Tukey’s multiple comparisons test to analyze the differences between each pair of groups. *P*-values of <0.05 were considered significant. Experiments were performed in triplicate and repeated at least two times.

## Results

### Physical Characterization of OMVs From *E. coli*

**Figure 1:**
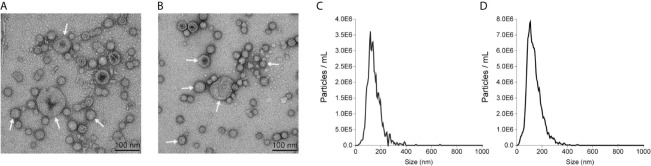
Representatives of transmission electron microscopy images of *E coli* 08-85 OMVs **(A)** and *E coli* K12 OMVs **(B)** recorded with negative staining with 2% phosphotungstic acid. The size distribution of OMVs of *E. coli* 08-85 **(C)** and *E coli* K12 OMVs **(D)** observed by nanoparticle tracking analysis. The OMVs were free of contaminating bacteria and the diameter ranged from 40 to 200 nm.

Our previous work identified a pathogenic *E. coli* strain (*E. coli* 08-85) carrying a *mcr-1* gene (Liang et al., 2021). *E. coli* ATCC25922 was selected as a control strain that is colistin sensitive and *mcr-1* negative. *E. coli* K12 and the isogenic *E. coli* 50434 (*E. coli* K12 with the *mcr-1* gene) were also used throughout this study. OMVs of these strains were purified. Transmission electron microscopy analysis revealed that these *E. coli* strains released OMVs at the late log-phase of growth. The vesicles were spherical and ranged in diameter from 50 to 200 nm (**Figures 1A, B**). The nanoparticle tracking analysis (NTA) of the OMVs determined that the median diameters were 134.2 and 124.9 nm for OMVs isolated from *E. coli* 08-85 and *E. coli* K12 respectively (**Figures 1C, D**). The size distribution of the vesicles was in general agreement by both methods.

### OMVs Protected a Susceptible *E. coli* Strain From Colistin

OMVs from clinical *E. coli* ATCC25922 and *E. coli* 08-85, as well as *E. coli* K12 and *E. coli* 50434, were purified, and the effect of OMVs on the bacterial growth and protection of killing from colistin was studied. The MICs of colistin were 1 μg/ml against *E. coli* ATCC25922 and *E. coli* K12, while the minimal bactericidal concentrations (MBCs) of colistin were 2 μg/ml against *E. coli* ATCC25922 and *E. coli* K12 (**Table 1**).

**Figure 2:**
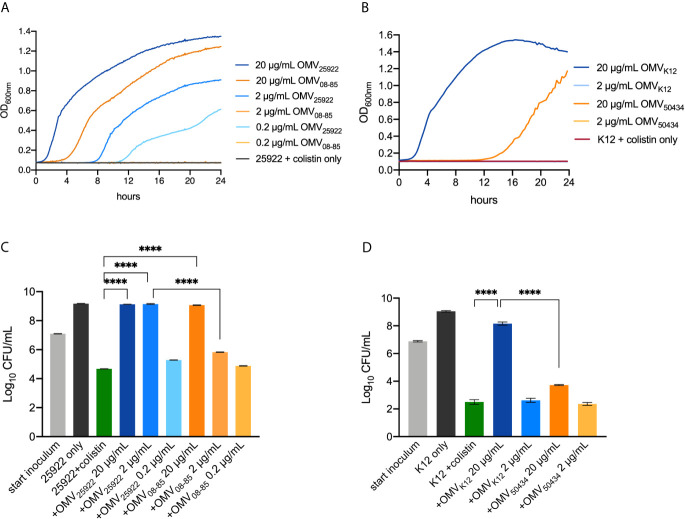
Growth curves **(A, B)** and killing protection assays **(C, D)** showed that OMVs of *mcr-1* positive *E coli* strains provided attenuated protection from colistin. **(A)** A dilution (1:1,000) of overnight culture of *E coli* ATCC25922 (colistin-susceptible) co-incubated with 20, 2, or 0.2 μg/ml OMVs isolated from *E. coli* ATCC25922 (*mcr-1* negative) or *E. coli* 08-85 (*mcr-1* positive) was treated with 2 μg/ml colistin. **(B)** A 1:1,000 dilution of overnight culture of *E. coli* K12 (colistin-susceptible) co-incubated with 20 or 2 μg/ml OMVs isolated from *E. coli* K12 (*mcr-1* negative) or *E. coli* 50434 (*mcr-1* positive) was treated with 2 μg/ml colistin. The turbidity was read every 15 min for 24 h. *E. coli* ATCC25922 without OMVs was set as the negative control. The concentration of OMVs tested in the killing assay was the same as the growth curve assay. Log-phase culture (10^7^ CFU/ml) of *E. coli* ATCC25922 **(C)** or *E. coli* K12 **(D)** was treated with colistin for 6 h, and the surviving bacterial cells were counted. The bacterial cells alone and bacterial cells with colistin alone were set as positive and negative controls respectively. Asterisks (*) denote statistical significance which was determined by one-way ANOVA followed by Turkey’s multiple-comparison test (****P < 0.0001, n = 3, bars represent mean ± s.e.m.).

*E. coli* ATCC25922 or *E. coli* K12, incubated with serial concentrations of OMVs ranging from 0.2 to 20 μg/ml, was treated with 2× MIC colistin (2 μg/ml) to ensure that the bacteria did not grow at this concentration. As shown in **Figures 2A, B**, the bacterial cells of *E. coli* ATCC25922 or *E. coli* K12 in the absence of OMVs did not grow at all. OMVs protected *E. coli* ATCC25922 from growth inhibitory in a concentration-dependent manner, with the bacterial cells growing fastest with 20 μg/ml OMVs and slowest or no growth with 0.2 μg/ml OMVs (**Figure 2A**). Notably, OMVs from the *mcr-1* positive strain *E. coli* 08-85 conferred less protection than OMVs isolated from *E. coli* ATCC25922 at the same concentration (**Figure 2A**). In the same way, *E. coli* K12 was protected by 20 μg/ml *E. coli* K12 OMVs, while the growth was totally inhibited within the first 12 h with 20 μg/ml *E. coli* 50434 OMVs (**Figure 2B**).

In the killing protection assays (**Figures 2C, D** **)**, the bacterial cells alone (black bars) and bacterial cells with colistin (2 μg/ml, green bars) were set as the positive and negative controls respectively. For clinical *E. coli* ATCC25922 (**Figure 2C**), both 20 μg/ml *E. coli* ATCC25922 OMVs and 20 μg/ml *E. coli* 08-85 OMVs provided protection from colistin treatment compared with the negative control. However, when incubated with 2 μg/ml OMVs, bacterial cells were completely protected with *E. coli* ATCC25922 OMVs, but not OMVs of *E. coli* 08-85. A 0.2 μg/ml OMVs of *E. coli* ATCC25922 or *E. coli* 08-85 was not able to protect *E. coli* ATCC25922 against colistin. As shown in **Figure 2D**, the colistin sensitive *E. coli* K12 was protected by 20 μg/ml *E. coli* K12 OMVs from colistin treatment compared with *E. coli* K12 without OMVs. However, the addition of 20 μg/ml *E. coli* 50434 OMVs conferred significantly attenuated protection against colistin. Neither 2 μg/ml *E. coli* K12 OMVs nor 2 μg/ml *E. coli* 50434 OMVs could protect *E. coli* K12 from colistin.

These results indicated that OMVs from the *mcr-1* positive strain attenuated the protective effect when compared to the *mcr-1* negative strain at the same concentration.

### The Protection Was Related to the Absorption of Colistin by OMVs

**Figure 3:**
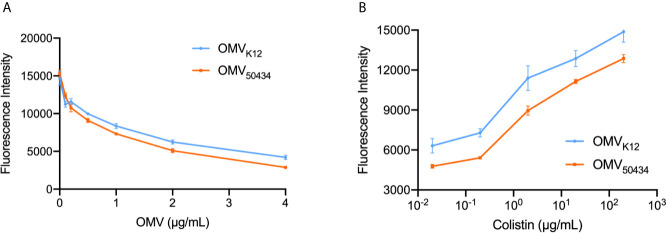
Titration of OMVs against BC **(A)** and displacement of OMV-bound BC by colistin **(B)**. **(A)** BCs (4 μM) were titrated by OMVs isolated from *E. coli* K12 or *E. coli* 50434 (*E. coli* K12 with *mcr-1*) ranging from 0.1 to 4 μg/ml protein concentration. **(B)** Colistin (0.02–200 μg/ml) was successively added to the BC : OMV complex. Excitation: 580 nm; Emission: 620 nm. n = 3.

It has been reported that OMVs could serve as decoys that absorb antibiotics and provide protection for sensitive bacteria (Manning and Kuehn, 2011). To test whether the protection of OMVs observed in the protection assay was due to the absorption of colistin by OMVs, a fluorescent displacement assay using BODIPY TR cadaverine (BC), which binds to lipid A, was used according to a previously described assay (Wood et al., 2004). The fluorescence of BC is quenched by binding to lipid A phosphate groups and de-quenched by compounds displaying an affinity for lipid A. OMVs isolated from *E. coli* K12 and *E. coli* 50434 were used for the following studies. Strain *E. coli* 50434 was engineered from *E. coli* K12 with the *mcr-1* gene introduced into the chromosome (Qiao, 2019). As shown in **Figure 3A**, the titration of OMVs against BC resulted in a concentration-dependent quenching of BC fluorescence. If colistin binds to the lipid A of OMVs similarly, it should displace the BC probe and lead to an increase of fluorescence intensity. Serial 10-fold dilutions of colistin were added to BC (4 μM) solution that was pre-incubated with 2 μg/ml OMVs of *E. coli* K12 or *E. coli* 50434. The fluorescence of BC increased while the concentration of colistin increased, which demonstrated that colistin binds to OMVs in a dose-dependent manner (**Figure 3B**).

NPN uptake analysis was also performed to determine the interaction of OMVs and colistin. NPN is a lipophilic probe with low fluorescence quantum yield in an aqueous environment and becomes fluorescent when partitioned in a hydrophobic environment (Helander and Mattila-Sandholm, 2000). The binding of colistin to OMVs results in increased membrane permeability and increased fluorescence of NPN. As shown in **Figure S1**, the fluorescence of NPN increased while the concentration of colistin increased, which indicated that the colistin was absorbed by OMVs.

Notably, when incubated with the same concentration of colistin, there was more BC de-quenched by *E. coli* K12 OMVs than OMVs of *E. coli* 50434 (**Figure 3B**). Similarly, the uptake of NPN by OMVs of *E. coli* 50434 was less than that of *E. coli* K12 OMVs (**Figure S1**). These results suggested OMVs of *E. coli* K12 absorbed more colistin than OMVs of *E. coli* 50434 at the same concentration and could explain the attenuated protection provided by OMVs of *mcr-1* positive *E. coli* strains. To assess if the difference in the ability to bind colistin was due to lipid A modification caused by the MCR-1 enzyme, the lipid A contents were then analyzed.

### The pEtN Modified Lipid A Was Present in the OMVs of a *mcr-1* Positive *E. coli* Strain

**Figure 4:**
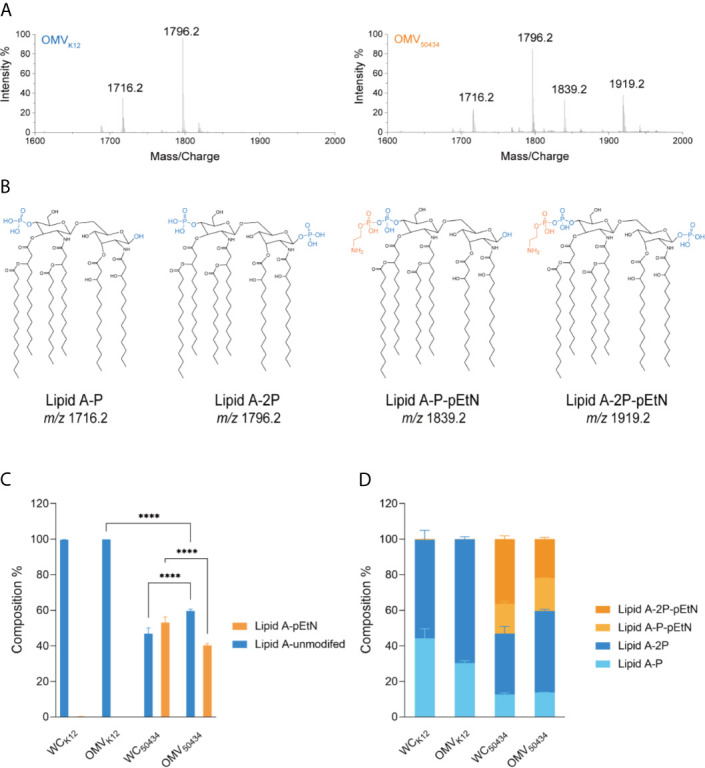
Characterization and relative quantitation of lipid A in OMVs and the producer whole cells (WCs) by LC-MS. **(A)** Representative mass spectra of lipid A from *E. coli* K12 OMVs (left) and *E. coli* 50434 OMVs (right). **(B)** Structures of lipid A species identified by mass spectrometry. Phosphate group, blue; pEtN, orange. **(C, D)** The composition of lipid A species was relatively quantitated of peak area with MultiQuant 3.0. Asterisks (*) denote statistical significance as determined by two-way ANOVA followed by Turkey’s multiple-comparison test (****P < 0.0001, n = 3).

Lipid A of OMVs purified from *E. coli* K12 and *E. coli* 50434 was isolated and identified with LC-MS. Two species of unmodified lipid A, 1 or 4′-phosphate (Lipid A-P, m/z of 1716.2) and 1,4-bisphosphate (Lipid A-2P, m/z of 1796.2) were confirmed to be present in both *E. coli* K12 and *E. coli* 50434 OMVs. A single pEtN attached to these unmodified lipid A, lipid A-P-pEtN (m/z of 1839.2), and lipid A-2P-pEtN (m/z of 1919.2) was observed in the OMVs of *E. coli* 50434 (**Figures 4A, B**).

The relative amounts of the lipid A species detected were calculated. The lipid A species with two phosphate groups were more abundant than that with only one phosphate group, whether modified with pEtN or not (**Figure 4D**). Interestingly, there was less pEtN-modified lipid A present in the OMVs compared with bacterial cells, with 46 and 60% in composition respectively (**Figure 4C**).

These results confirmed that the pEtN-modified lipid A was present in the OMVs of the *mcr-1* positive *E. coli* strain. This could explain why OMVs of the *mcr-1* positive strain conferred attenuated protection compared with OMVs of the *mcr-1* negative strain at the same concentration. Interestingly, we noticed that the relative amounts of pEtN-modified and native lipid A between bacterial cells and OMVs of *E. coli* 50434 were not identical; the unmodified lipid A was more likely to be secreted into OMVs, and the pEtN modified lipid A was enriched in bacterial cells.

### An *E. coli* Strain Carrying the *mcr-1* Gene Produced More OMVs

**Figure 5:**
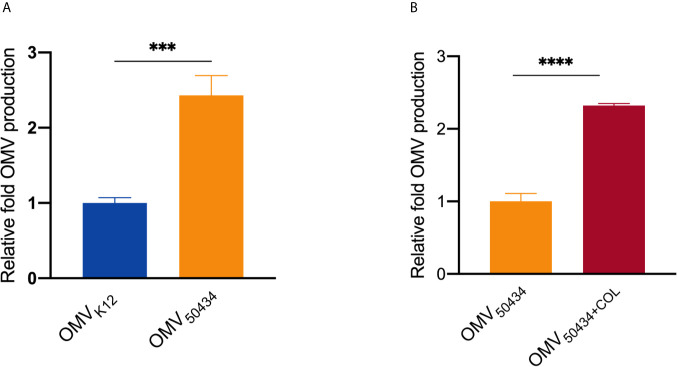
Relative quantitation of OMV production. OMV production was normalized to CFU counts and quantitated for lipid content by FM4-64. **(A)** The OMV production of *E. coli* K12 *vs E. coli* 50434 (*E. coli* K12 with *mcr-1*). **(B)** The OMV production of *E. coli* 50434 grown with or without colistin (COL, 2 μg/ml). Asterisks (*) denote statistical significance as determined by unpaired t-test (***P < 0.001, ****P < 0.0001, n = 3, bars represent mean ± s.e.m.)

Finally, we investigated the impact of the *mcr-1* gene on OMV production. The relative concentrations of OMVs from *E. coli* K12 or *E. coli* 50434 were determined by measuring lipid content *via* fluorescence from the lipophilic dye FM4-64 (McBroom et al., 2006). The OMV production was normalized by dividing by the number of CFU/ml, and the relative fold OMV production was calculated as these values were further divided by the OMV production of the control condition. Compared with *E. coli* K12, the *mcr-1* positive *E. coli* 50434 released 2.5-fold more OMVs (**Figure 5A**). For strain *E. coli* 50434, growing with colistin pressure (2 μg/ml) resulted in a significant increase of OMV production (2.3-fold more) compared with *E. coli* 50434 growing without colistin (**Figure 5B**).

## Discussion

Resistance to colistin, especially the recently reported plasmid-mediated colistin resistance, is a serious threat to global public health since the distribution of these plasmids among Gram-negative bacterial may be a further step towards the “pre-antibiotic era” (Bialvaei and Samadi Kafil, 2015). Thus, understanding the mechanism of colistin resistance is of great significance for developing new strategies against colistin-resistant bacteria. Previous reports have shown that OMVs mediated antibiotic resistance mainly in two ways: a) horizontally transferring antibiotic resistance gene (*e.g.* the extended spectrum βD;-lactamase gene *bla_CTX-M-15_* (Bielaszewska et al., 2020), the New Delhi metallo-β-lactamase-1 gene *bla_NDM-1_* (Chatterjee et al., 2017), and the β-lactamase gene *bla_OXA-24_* (Rumbo et al., 2011)); b) reducing the antibiotic concentration in the environment either by directly absorbing it (e.g. membrane-active antibiotics) (Manning and Kuehn, 2011; Kulkarni et al., 2015) or packing antibiotic resistance enzymes in their lumen (*e.g.* β-lactamase) (Lee et al., 2013). So far, little is known about the role of OMVs in *mcr-1* mediated colistin resistance. It is not surprising to speculate that OMVs may participate in the horizontal transfer of the *mcr-1* gene which is carried on a plasmid. The *mcr-1* gene was detected in the OMVs of *E. coli* 08-85 by dot blotting and PCR (**Figure S2** and **Figure S3A**). Although there was no plasmid detected in *E. coli* 08-85 OMVs by pulsed-field gel electrophoresis (PFGE) digested with S1-nuclease (**Figure S3B**), we do not know whether the presence of the *mcr-1* gene in *E. coli* 08-85 OMVs was associated with the plasmid or not since the PFGE method is not sensitive enough. However, the bacterial cells of *E. coli* ATCC25922 that survived the colistin treatment in the killing protection assay were collected, and the *mcr-1* gene was not detected in any of them by PCR (**Figure S4**). Thus, it seems likely that the protective effect conferred by OMVs is not associated with the acquisition of the *mcr-1* gene from *E. coli* 08-85 OMVs.

Manning and Kuehn (2011) and Kulkarni et al. (2015) showed that the addition of purified OMVs to the *E. coli* cultures provided substantial protection against antimicrobial peptides (AMPs). Moving a step forward on their research, we found that OMVs of a *mcr-1* positive strain attenuated the effect of protection. Consistent with their study, we showed that OMVs, both from the *mcr-1* positive and negative *E. coli* strains, conferred dose-dependent protection against colistin sensitive *E. coli* strains. However, the addition of 2 μg/ml *E. coli* ATCC25922 (*mcr-1* negative) OMVs achieved 100% protection to the producer organism from colistin treatment, while 2 μg/ml *E. coli* 08-85 (*mcr-1* positive) OMVs could not protect the colistin sensitive strain *E. coli* ATCC25922 (**Figure 2C**). We noticed that the protective effects of 2 μg/ml *E. coli* ATCC25922 OMVs and 20 μg/ml *E. coli* 08-85 OMVs in the growth curve assay were not exactly the same as in the killing protection assay. This could be due to the inoculum effect in which the initial density of cells affects the activity of many antibiotics including membrane-active peptides (Loffredo et al., 2021). The growth curve assay was started with a 1:1,000 dilution of the overnight culture (starting inoculum of 10^5^ CFU/ml), while the log-phase culture was used as the starting inoculum which was 10^7^ CFU/ml in the killing protection assay. Colistin was absorbed by OMVs (**Figure 3**), and there should be less free colistin left in the environment for killing bacterial cells after co-incubation with 20 μg/ml *E. coli* ATCC25922 OMVs than 2 μg/ml *E. coli* ATCC25922 OMVs. When incubated with 20 μg/ml *E. coli* ATCC25922 OMVs, the free colistin left might not be enough to either inhibit the growth or kill bacterial cells. However, when co-incubated with 2 μg/ml *E. coli* ATCC25922 OMVs or *E. coli* 08-85 OMVs, the free colistin left in the environment might be able to inhibit the growth of bacterial cells at a 10^5^ CFU/ml inoculum, but might not be enough to kill an inoculum of 10^7^ CFU/ml due to the inoculum effect. Also, we noticed that there were still 10,000 viable bacteria of colistin (2× MIC) treated *E. coli* ATCC25922 in the killing protection assays (**Figure 2C**). This could be due to the inoculum effect as well since the MBCs were determined with the inoculum of 10^5^ CFU/ml (according to CLSI), while the starting inoculum was 10^7^ CFU/ml in the killing protection assay. The attenuated protective effect was also observed in *E. coli* K12 and the isogenic strain *E. coli* 50434 when incubated with 20 μg/ml OMVs. LPSs are complex molecules that consist of O antigen, the core oligosaccharide and the lipid A moiety. The differences in genetic background between *E. coli* ATCC25922 and *E. coli* K12 might result in the different structure of LPS and the bacterial outer membrane, thus leading to the different binding affinity of OMVs to colistin (McInerney et al., 2016). The OMVs of *E. coli* ATCC25922 might have a better binding affinity to colistin than *E. coli* K12 OMVs. Therefore, 2 μg/ml of *E. coli* K12 OMVs might not be enough to protect from 2 μg/ml colistin due to the strain specificity, and this could be the reason for the relatively weaker protection provided by *E. coli* K12 OMVs compared with *E. coli* ATCC25922 OMVs, especially at 2 μg/ml.

MCR-1 catalyzes the transfer of pEtN to lipid A and decreases colistin affinity. Thus, we wondered if the protection of OMVs observed in the protection assay was due to the absorption of colistin by OMVs. If that was the case, the attenuated protection of OMVs from the *mcr-1* positive strain could be related to the reduced ability to absorb colistin which is caused by the modification of lipid A. To address this, we first determined the ability of OMVs to bind to colistin. Since *E. coli* ATCC25922 and clinical isolate *E. coli* 08-85 are two strains with different genetic backgrounds, *E. coli* K12 and *E. coli* K12 recombineered with the *mcr-1* gene (*E. coli* 50434) were used for the molecular mechanism studies. To avoid the interference of the potential horizontal transfer of *mcr-1* genes, strain *E. coli* 50434 was engineered with the *mcr-1* gene introduced into the chromosome. We confirmed that colistin was absorbed by OMVs by both the BC replacement (**Figure 3**) and NPN uptake assays (**Figure S1**). In both methods, *E. coli* 50434 OMVs showed less ability to bind to colistin, and this is consistent with the attenuated protection of *mcr-1* positive strain OMVs exhibited in **Figure 2**.

We confirmed that *E. coli* 50434 OMVs contained pEtN-modified lipid A molecules, while lipid A in *E. coli* K12 OMVs was unmodified. This explained the observation of the attenuated protective effect and binding affinity to colistin of OMVs from the *mcr-1* positive strain compared to the negative strain (**Figures 2**, **3**). For the *mcr-1* positive strain, when comparing the composition of lipid A species between *E. coli* 50434 OMVs and its producer cells, we found that they packed more unmodified lipid A in OMVs and retained more pEtN modified lipid A in the outer membrane of bacterial cells. Both the colistin-sensitive strain and themselves could benefit from this arrangement as they release OMVs with more unmodified lipid A for better protection for sensitive strains and left more pEtN-modified lipid A for themselves to combat against colistin. Several investigations have shown that OMV biogenesis is a deliberate process with cargo selection (Ayalew et al., 2013; Bonnington and Kuehn, 2014). Our results provided further support to this phenomenon by adding evidence of adjustment of lipid A species in OMVs of the *mcr-1* positive *E. coli* strain for combating colistin and causing and spreading resistance to colistin. Nevertheless, it should be noted that the *E. coli* strain with *mcr-1* released more OMVs than *E. coli* K12 and two-fold more under colistin pressure. The mechanism of increased OMV production of the *mcr-1* positive strain is unclear and needs further investigation. Our work, for the first time, sheds light on how *mcr-1* affected the lipid contents of OMVs and added new insights into the research field of OMVs and antibiotic resistance.

## Conclusion

Our study found that OMVs from the *mcr-1* positive strain conferred attenuated protection against colistin when compared to OMVs from the *mcr-1* negative strain. This could be due to the presence of pEtN-modified lipid A in the OMVs of *mcr-1* positive *E. coli* strain, which resulted in the decreased ability to absorb colistin in the environment.

## Data Availability Statement

The original contributions presented in the study are included in the article/**Supplementary Material**. Further inquiries can be directed to the corresponding authors.

## Author Contributions

YZ and XL conceived and designed the study. XL, YZ, LS, XW, XH, and TN carried out the experiments and analyzed the data. CL and XYa revised the manuscript. XYo is the director of this work and responsible for the general supervision of the study. All authors contributed to the article and approved the submitted version.

## Funding

This research was funded by the National Natural Science Foundation of China (81803413), the Peking Union Medical College Youth Fund (PUMC) (3332018093), the National Mega-project for Innovative Drugs (2019ZX09721001), the CAMS Initiative for Innovative Medicine (2016-I2M-3- 014).

## Conflict of Interest

The authors declare that the research was conducted in the absence of any commercial or financial relationships that could be construed as a potential conflict of interest.

## Publisher’s Note

All claims expressed in this article are solely those of the authors and do not necessarily represent those of their affiliated organizations, or those of the publisher, the editors and the reviewers. Any product that may be evaluated in this article, or claim that may be made by its manufacturer, is not guaranteed or endorsed by the publisher.
