# Supplementary material for: The Attenuated Protective Effect of Outer Membrane Vesicles Produced by a mcr-1 Positive Strain on Colistin Sensitive Escherichia coli
Source: Front Cell Infect Microbiol. 2021 Jul 28;11:701625. doi: 10.3389/fcimb.2021.701625 (PMC8355893; doi:10.3389/fcimb.2021.701625)
Supplement: Supplementary file 1 [file DataSheet_1.pdf]

Supplementary Material

Supplementary Data

N-Phenyl-1-naphthylamine (NPN) uptake assay

To investigate the interaction between OMVs and colistin, NPN uptake assays were conducted as previously established [1, 2]. Briefly, OMVs (from *E. coli* K12 and *E. coli* 50434), colistin, and NPN were diluted with 5 mM HEPES buffer containing 20 mM glucose. A volume of 100 µL of OMVs (final concentration of 5 µg/mL) was added to 50 µL of buffer containing NPN (final concentration of 10 µM) and varying concentrations of colistin (final concentration of 6.25 to 0.25 mg/mL) in black 96-well plates. The fluorescence was read with excitation 355 ± 5 nm and emission 420 ± 5 nm. 1% Triton X-100 was used as positive control and OMVs with NPN in the absence of colistin were set as negative control (F_0). Percent NPN uptake is calculated as follow: NPN uptake (%) = $(F - F_0) / (F_{\text{triton X100}} - F_0) \times 100\%$.

PCR

The primers and procedure for detecting the *mcr-1* gene in OMVs and bacterial cells are the same as previously described [3]. Primers used were: MCR-1 2Fs F: 5'-ATGATGCAGCATACTTCTG-3'; MCR-1 2Fs R: 5'-TCAGCGGATGAATGCGGTG-3'. 16s 27F: 5'-AGAGTTTGATCCTGGCTCAG-3'; 16S 1492R 5'- GGTTACCTTGTTACGACTT-3'.

Dot blotting

The dot blotting assay was performed according to a previous study with small modifications [4]. The DNA probe was made by amplifying the *mcr-1* gene from *E. coli* 08-85 and labeled with DIG-High Prime. Samples were heat-denatured at 96 ˚C for 10 min, cooled on ice and spotted onto an Immobilon-NY+ membrane (Millipore). The membrane was cross-linked by UV irradiation and pre-hybridized with DIG Easy Hyb (10 ml/100 cm^2). The DNA probe was then added and incubated overnight at 60 ˚C. The membrane was washed twice with 2×SSC (containing 0.1 %SDS) and then blocked with maleic acid for 30 min and incubate with Anti-Digoxigenin-AP conjugate at 1:5000 dilution. The unbounded conjugates were washed off and 200 µL of NBT/BCIP substrate were added to the membrane and the image was acquired by Gel Doc XR+ (bio-rad).

S1-PFGE

The presence of plasmid in OMVs and bacterial cells were examined using PFGE with S1 nuclease treatment (S1-PFGE) according to the previously reported protocol [3]. Briefly, bacterial cells and OMVs were prepared in agarose blocks and digested with S1 nuclease for 15 min at 23˚C. *Salmonella serotype Braenderup* H9812 digested with XbaI (37 ˚C for 3h) was used as the marker. The plasmids were separated using the CHEF DRII system (Bio-Rad, Hercules, CA) for 22h (6 V/cm and 14˚C) with initial and final pulses conducted for 5 and 25 sec. The image was visualized with Gel Doc XR+ (bio-rad).

Supplementary Figures

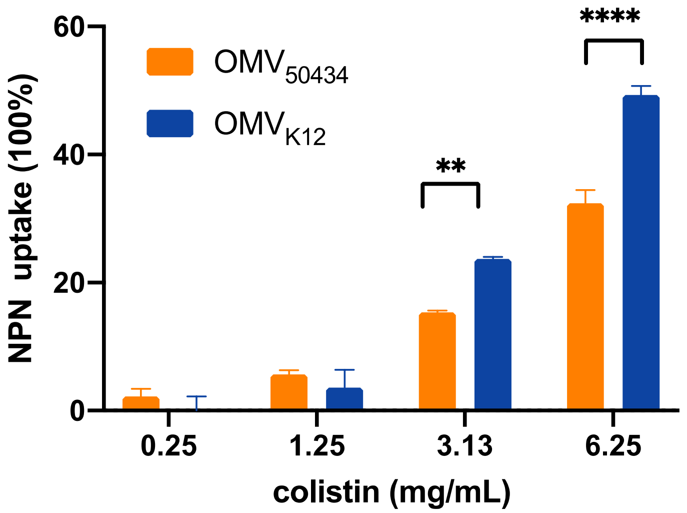

Figure S1. N-Phenyl-1-naphthylamine (NPN) uptake of *E. coli* K12 OMVs and *E. coli* 50434 OMVs. Asterisks (*) denote statistical significance as determined by two-way ANOVA followed by Turkey's multiple-comparison test (**, $P < 0.01$, ****, $P < 0.0001$, n = 3)

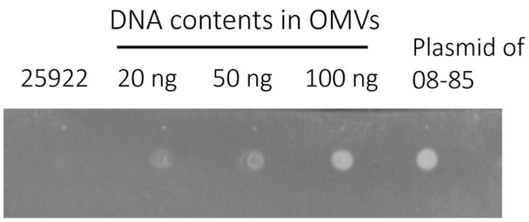

Figure S2. The *mcr-1* gene was presented in OMVs of *E. coli* 08-85 as detected by dot blotting.

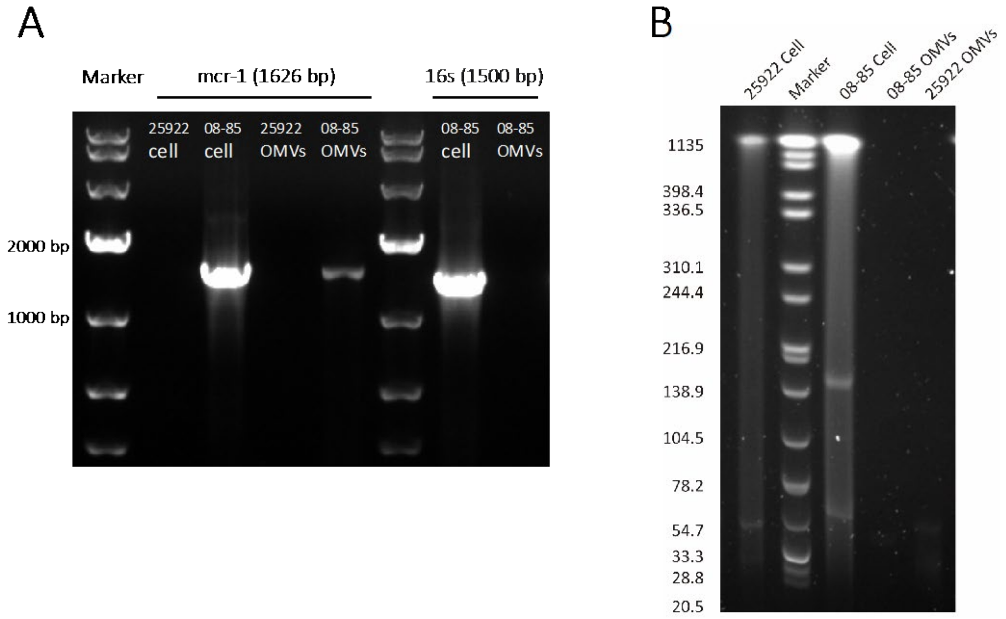

Figure S3. (A) PCR of *mcr-1* and 16s genes of OMVs and bacterial cells. (B) Plasmids were not detected in *E. coli* 08-85 OMVs examined by S1-PFGE. *Salmonella serotype Braenderup* H9812 digested with XbaI (37 ˚C for 3h) was used as the marker.

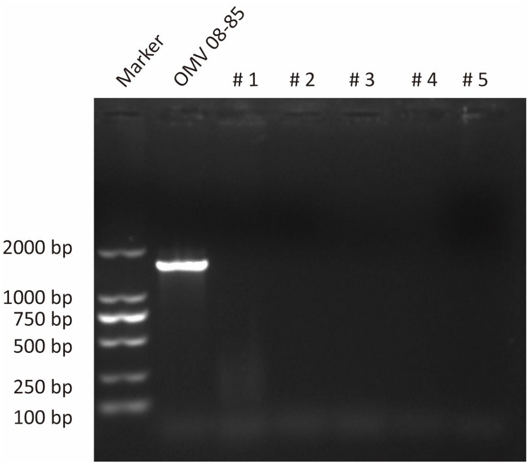

Figure S4. *mcr-1* gene was not detected in *E. coli* ATCC25922 protected by OMVs from colistin. #1 ~ #5 are representatives of ATCC25922 colonies protected by *E. coli* 08-85 OMVs from colistin treatment.

References

1. Kulkarni, H.M., R. Nagaraj, and M.V. Jagannadham, *Protective role of E. coli outer membrane vesicles against antibiotics.* Microbiological research, 2015. **181**: p. 1-7.

2. Helander, I. and T. Mattila - Sandholm, *Fluorometric assessment of Gram - negative bacterial permeabilization.* Journal of applied microbiology, 2000. **88**(2): p. 213-219.
3. Liang, Z., J. Pang, X. Hu, T. Nie, X. Lu, X. Li, et al., *Low Prevalence of mcr-1 Among Clinical Enterobacteriaceae Isolates and Co-transfer of mcr-1 and bla NDM-1 from Separate Donors.* Microbial Drug Resistance, 2021. **27**(4): p. 476-484.
4. Rumbo, C., E. Fernández-Moreira, M. Merino, M. Poza, J.A. Mendez, N.C. Soares, et al., *Horizontal transfer of the OXA-24 carbapenemase gene via outer membrane vesicles: a new mechanism of dissemination of carbapenem resistance genes in Acinetobacter baumannii.* Antimicrobial agents and chemotherapy, 2011. **55**(7): p. 3084-3090.
